# Electromagnetic field effect on weak-coupling piezoelectric polaron in an asymmetrical Gaussian confinement potential quantum well

**DOI:** 10.1016/j.heliyon.2022.e10505

**Published:** 2022-09-06

**Authors:** F. Manfouo, I. Nsangou, M.F.C. Fobasso, A.J. Fotue

**Affiliations:** Mesoscopic and Multilayers Structures Laboratory, Department of Physics, Faculty of Science,University of Dschang, P.O. Box 479 Dschang, Cameroon

**Keywords:** Piezo-electric polaron, Ground state energy, Ground state binding energy, Quantum well

## Abstract

The properties of an electron weakly coupled to piezo-acoustic phonon in asymmetrical Gaussian confinement potential quantum well (AGCPQW) subject to external electric field (EF) and magnetic field (MF) has been investigated using the Lee-Low-Pines unitary transformation and linear combination operation methods. We have obtained the ground state energy (GSE) and the ground state binding energy (GSBE) of piezoelectric polaron. The effects of the EF, the MF, the range of the asymmetrical Gaussian confinement potential (RAGCP), Debye cut-off wavenumber (DCOW) and the electron–phonon coupling strength on the GSE and the GSBE are also analyzed. It is found that the GSE is an increasing function of the EF and the cyclotron frequency (CF), whereas it is a decreasing one of the RAGCP, the DCOW and electron–phonon coupling strength. The GSBE is an increasing function of the DCOW and the electron–phonon coupling strength. It is also an aggrandizing function with decreasing of the RAGCP, whereas it is a decayed one of the EF and CF. It is shown that the EF, the RAGCP, the MF, the DCOW and electron–phonon coupling strength are important factors that have great influence on the properties of the piezoelectric polaron in AGCPQW.

## 1 Introduction

With the progress of nanotechnology, it is now possible to fabricate a variety of small-sized nano-materials such as quantum well (QW), quantum dot (QD), quantum wires [1, 2, 3, 4] in which the electron change their behavior. These nanostructures display a lot of powerful physical properties that have found a great potential application in telecommunication, micro-electronic devices, solar cells, quantum computers etc [1, 3]. The properties of confined electron become more suitable in the presence of MF and EF. Consequently, many scientists devoted their investigations in the physical properties of small-sized quantum systems in the presence of external field [5, 6, 7, 8, 9]. QW with Gaussian potential is an effective theoretical model for describing real case, and has been extensively demonstrated by some theoretical and practical works in the last two decades [10, 11, 12, 13, 14]. There are also important other investigations dealing with different types of confinement potentials the hyperbolic potential [15], cylindrical potential [16] and parabolic potential [17]. It is well-known that the electron–phonon interaction should influence a lot the properties of quantum system and therefore has been widely studied. The quasiparticule that emerges from the coupled electron-phonon is call polaron [18]. The influence of polaron inside low-dimensional nano-materials is more important thus, it have received a great attention in recent years [19, 20, 21]. In crystal without an inversion center or piezoelectric crystal, a conduction electron interacts with the acoustic phonon and form the piezoelectric polaron [18, 22, 23]. Some materials are excellent candidate for low-dimensional quantum systems like ZnO, GaN, CdS and GaAs [23, 24]. Those materials are a suitable weak-coupling polar piezoelectric semiconductors in which the polaron affect drastically the properties of the nanostructures [24, 25, 26, 27, 28, 29]. The piezoelectric polaron has been the subject of much discussion in recent years. The importance of electron-piezo-acoustic phonon interaction in crystal was reported by Hutson [23]. He determined approximate values of the mobility of piezoelectric polaron and also discussed about Seebeck effect in ZnO due to electron-piezo-acoustic phonon interaction and many experiments followed. Matsuura and Wang [30] calculated the GSE of a bound piezoelectric polaron by using the perturbation theory. Parker and Whitfield [31] obtained an energy-momentum relation for the moving piezoelectric polaron by using the strong coupling polaron theory. Tokuda [32] calculated the energies and effective mass of the optical and the piezoelectric polarons in weak coupling limit by using the method that bears his name. Rona and Whitfield [33] investigated the energy-momentum relation for the piezoelectric polaron by using the intermediate-coupling theory. Shoji and Tokuda [34] used Huybrechts-like variational approach to calculate the GSE of piezoelectric polaron and another types of polaron in the weak and strong-coupling limits. They have also examined the phase-transition-like behaviour in different types of polaron. Licari and Whitfield [35], following the intermediate-coupling theory, examined anisotropic piezoelectric polaron. They demonstrated that the piezoelectric polaron has a maximum velocity in each direction. They have also evaluated the increase in effective mass due to the piezoelectric polaron effect for slow polarons. Several researches have been oriented on the behavior of the piezoelectric polaron under the MF. In 1970, Porsch [36] calculated the GSE and longitudinal effective masses of optical and piezoelectric polaron in presence of MF in strong-coupling limit. Klyukanov and Pokatilov [37] studied the thermodynamic functions of piezoelectric polaron and Cyclotron Resonance at weak and strong MF. Choi and Fujita [38] employing the Fujita's diagram method and Kubo's formula to examine the MF dependence of cyclotron resonance line width due to electron-acoustic phonon interactions in the extreme quantum limit. They found that the cyclotron resonance line width for all acoustic polarons increases with increasing field intensity. Pastor and Sadowski [39] analyzed the effect of electron-piezo-acoustic phonon interaction on the cyclotron resonance half-width in weakly polar semiconductors by employing the method given by Srinivas et al [40]. Using the Lee-Low-Pines unitary transformation and linear combination operation methods, Xinjun et al [13] in 2015 studied the influence of MF on the vibrational frequency, GSE and GSBE of a weak-coupling polar optical polaron in GaAs AGCPQW. However the influences of the external EF, MF, the Debye cut-off wavenumber (DCOW) on the weak coupling piezoelectric polaron in the AGCPQW have not yet received much attention. Recently, it was shown explicitly that the piezoelectric polaron is another important problem in the crystal. His form is essentially the same form as Fröhlich polaron [18].

In this paper, we intend to study the effects of the external EF, the range of the asymmetrical Gaussian confinement potential (RAGCP), the MF and DCOW on the GSE and the GSBE of weak coupling piezoelectric polaron by using the Lee-Low-Pines unitary transformation and linear combination operation methods. The paper is structured as follows: in section 2, we will present the theoretical model where the Hamiltonian of our system and the modified Lee-Low-Pines transformation method are described. In section 3, the numerical results are presented and discussed. The last section is devoted to the conclusion.

## 2 Theoretical and model

We consider the system in which the electron is confined in AGCPQW. The electron is moving in piezoelectric crystal and interacting with longitudinal piezoelectric-phonons in the presence of EF F applied along x-direction and parallel MF along the z direction with vector potential *A*. The study of this system is done by the Hamiltonian:

(1) $$H=\frac{1}{2m}{\left(P+\frac{eA}{c}\right)}^{2}+V\left(z\right)+exF+\sum_{q}\hbar \omega_{q}a_{q}^{+}a_{q}+{\left(\frac{4\pi \alpha }{V}\right)}^{\frac{1}{2}}\hbar s\sum_{q}\frac{1}{\sqrt{q}}\left(a_{q}+a_{-q}^{+}\right)e^{iq.r}$$

The first term in Eq. (1) describes the electron momentum energy, the second is the confining potential, the third term denotes the contribution of the external EF to the Hamiltonian, the fourth term represents the energy of acoustic lattice vibrations and the fifth term gives the electron-piezo-acoustic phonon interaction energy. Here, m is the electron band mass,e>0 is the elementary charge, $\omega_{q}=s.q$ is the linear dispersion which is used for the frequencies of piezo-acoustic phonons with a DCOW $q_{0}$ and s the velocity of sound. $a_{q}^{+}$ and $a_{q}$ are the creation and destruction operators for acoustic phonon of the wave vector q. P and r=(x,y,z) are momentum and position of electron. The piezoelectric coupling constant is given by:

(2) $$\alpha =\frac{1}{2}\frac{e^{2}\left\langle e_{ijk}^{2}\right\rangle }{\varepsilon^{2}Cs}.$$

In Eq. (2), $\left\langle e_{ijk}^{2}\right\rangle$ is an average of the piezoelectric tensor [32], ε is the dielectric constant, C is an average elastic constant. The confining potential in z-direction is giving by [13]:

(3) $$V\left(z\right)=\left\{ \begin{array}{ll} -V_{0}\;\exp\left(-\frac{z^{2}}{2R}\right) & z\geq 0\\ \infty & z<0 \end{array}\right..$$

In Eq. (3), $V_{0}$ is the height of AGCPQW and R is the RAGCP.

Employing the gauge approximation [41], the Hamiltonian can be express as:

(4) $$\begin{array}{l} H=\frac{1}{2m}P^{2}+\frac{m\omega_{c}^{2}}{8}\left(x^{2}+y^{2}\right)+\frac{\omega_{c}}{2}L_{z}+V\left(z\right)+exF\\ \;+\sum_{q}\hbar \omega_{q}a_{q}^{+}a_{q}+{\left(\frac{4\pi \alpha }{V}\right)}^{\frac{1}{2}}\hbar s\sum_{q}\frac{1}{\sqrt{q}}\left(a_{q}+a_{-q}^{+}\right)e^{iq.r} \end{array}$$

Where $\omega_{c}$ is the cyclotron frequency (CF) given by $\omega_{c}=\frac{eB}{m}$ with (c=1)

For simplicity, we have chosen units such as ℏ=m=s=e=1. Performing the Modified Lee-Low-Pines transformation [13] on the system, we introduce the first giving by the following Eq. (5), and second unitary transformation giving by following Eq. (6) to eliminate the electron and phonon coordinates.

(5) $$U_{1}=\exp\left\{-i\sum_{q}q.ra_{q}a_{q}^{+}\right\}$$

and

(6) $$U_{2}=\exp\sum_{q}\left(f_{q}a_{q}^{+}-f_{q}^{*}a_{q}\right)$$

Where $f_{q}\left(f_{q}^{*}\right)$ is the variational function. By using this two unitary transformations to Eq. (4), we can rewrite the Hamiltonian in the following form

(7) $$H^{\prime }=U_{2}^{-1}U_{1}^{-1}HU_{1}U_{2}$$

By minimizing the expectation value of the Hamiltonian (7), the GSE now is evaluated by the following Eq. (8) as:

(8) $$E_{0}=\left\langle \psi_{0}\right|\left\langle 0\right|H^{\prime }\left|0\right\rangle \left|\psi_{0}\right\rangle$$

Where $\psi_{0}$ is the electronic part of the ground-state wave function of the system and |0⟩ is the zero phonon state, which satisfies

(9) $$a_{q}\left|0\right\rangle =0\;\text{and}\;\left\langle \psi_{0}|\psi_{0}\right\rangle =\left\langle 0|0\right\rangle =1$$

We have used the relation

(10) $$\sum_{q}q{\left|f_{q}\right|}^{2}=0\;$$

Which comes from the total momentum conservation [42].

(11) $$\left\langle \psi_{0}\right|\left\langle 0\right|{\left(U_{2}U_{1}\right)}^{-1}\left(P+\sum_{q}qa_{q}^{+}a_{q}\right)\left(U_{1}U_{2}\right)\left|0\right\rangle \left|\psi_{0}\right\rangle =0$$

Choosing $\psi_{0}$ in the form:

(12) $$\left|\psi_{0}\right\rangle ={\left(\pi \right)}^{-3/4}\lambda^{3/2}\;\exp\left\{-\frac{\lambda^{2}}{2}\rho^{2}\right\}\exp\left\{-\frac{\lambda^{2}}{2}z^{2}\right\}$$

We can determine λ using a variational calculation. After performing Eqs. (9), (10), (11), and (12), the variational energy then simplifies to:

(13) $$\begin{array}{l} E_{0}=\left\langle \psi_{0}\right|\left(\frac{P^{2}}{2}+V\left(z\right)+xF\right)\left|\psi_{0}\right\rangle +\frac{{\omega_{c}}^{2}}{8}\left\langle \psi_{0}\right|\left(x^{2}+y^{2}\right)\left|\psi_{0}\right\rangle +\frac{\omega_{c}}{2}\left\langle \psi_{0}\right|L_{z}\left|\psi_{0}\right\rangle \\ \;+\;\sum_{q}\left(q+\frac{q^{2}}{2}\right){\left|f_{q}\right|}^{2}\;+{\left(\frac{4\pi \alpha }{V}\right)}^{\frac{1}{2}}\sum_{q}\frac{1}{\sqrt{q}}\left(f_{q}+f_{-q}^{*}\right) \end{array}$$

Minimzing the GSE giving by Eq. (13) with respect to the variational function $f_{q}$ yields and replacing summation $\sum_{q}$ into the integral $\left[V/4\pi^{3}\right]\int dq$ upper-limit $q_{0}$, we can easily obtain the polaron GSE in AGCPQW written as:

(14) $$E_{0}=\frac{3}{4}\lambda^{2}+\frac{{\omega_{c}}^{2}}{8\lambda^{2}}-V_{0}{\left(1+\frac{1}{2\lambda^{2}R^{2}}\right)}^{-\frac{1}{2}}+\frac{\sqrt{\pi }}{2\lambda }F-\frac{2\alpha }{\pi }\ln\left(1+\frac{q_{0}}{2}\right)$$

The variation of Eq. (14) with respect to λ gives

(15) $$\lambda^{4}-\frac{V_{0}}{3R^{2}}{\left(1+\frac{1}{2\lambda^{2}R^{2}}\right)}^{-3/2}-\frac{\sqrt{\pi }}{3}\lambda F-\frac{\omega_{c}^{2}}{6}=0$$

After solving Eq. (15) and supposing $E_{e}$ and $E_{ph}$ refers respectively to the energies of the independent electron and phonon, then the GSBE $E_{b}$ can be obtained following the formula [43].

(16) $$\begin{array}{l} E_{b}=E_{e}+E_{ph}-E_{0}\\ \;=\frac{4\alpha }{\pi }\ln\left(1+\frac{q_{0}}{2}\right)-\frac{{\omega_{c}}^{2}}{8\lambda^{2}}+V_{0}{\left(1+\frac{1}{2\lambda^{2}R^{2}}\right)}^{-1/2}-\frac{\sqrt{\pi }}{2\lambda }F \end{array}$$

## 3 Numerical results and discussions

**Figure 1:**
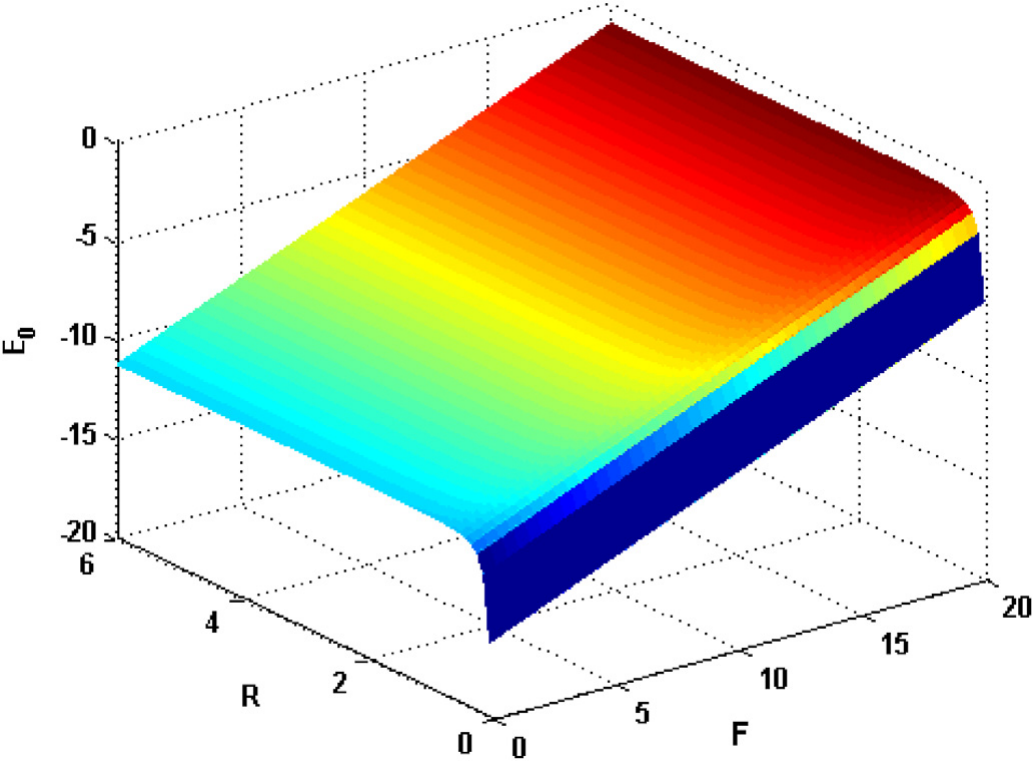
Relational curves of the GSE $E_{0}$ with RAGCP R and EF F for $q_{0}=50$, $\omega_{c}=5$$V_{0}=4$ and α=0.1.

**Figure 2:**
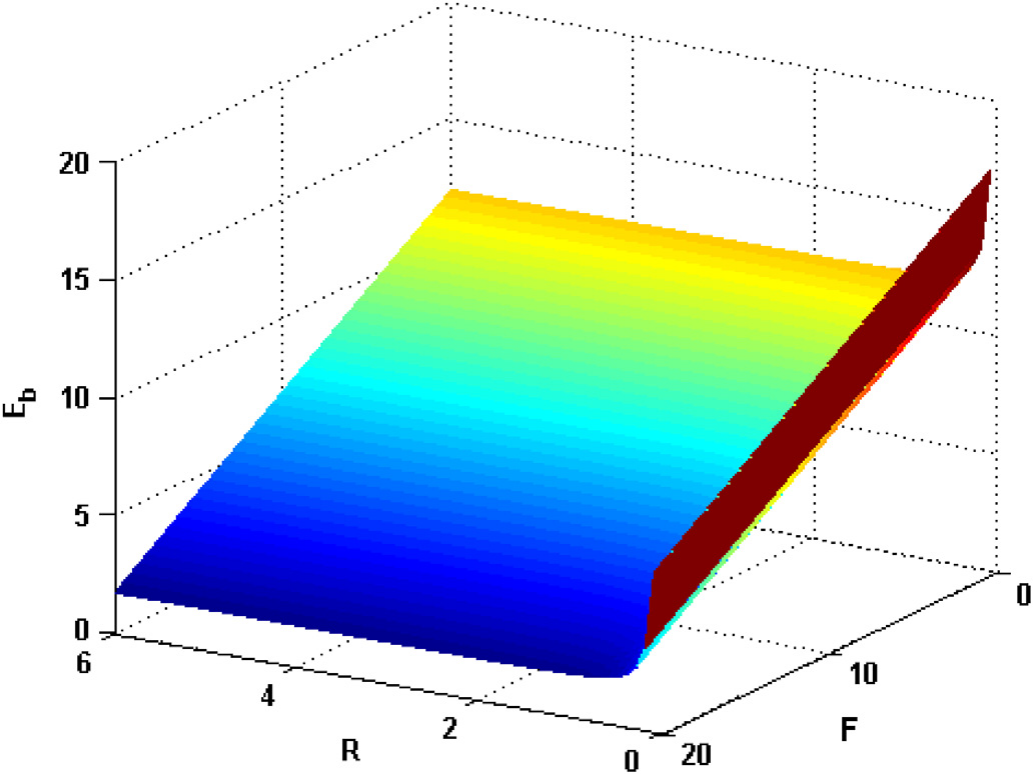
Relational curves of GSBE $E_{b}$ with RAGCP R and EF F for $q_{0}=50$ , $\omega_{c}=5$ , $V_{0}=4$ and α=0.1.

**Figure 3:**
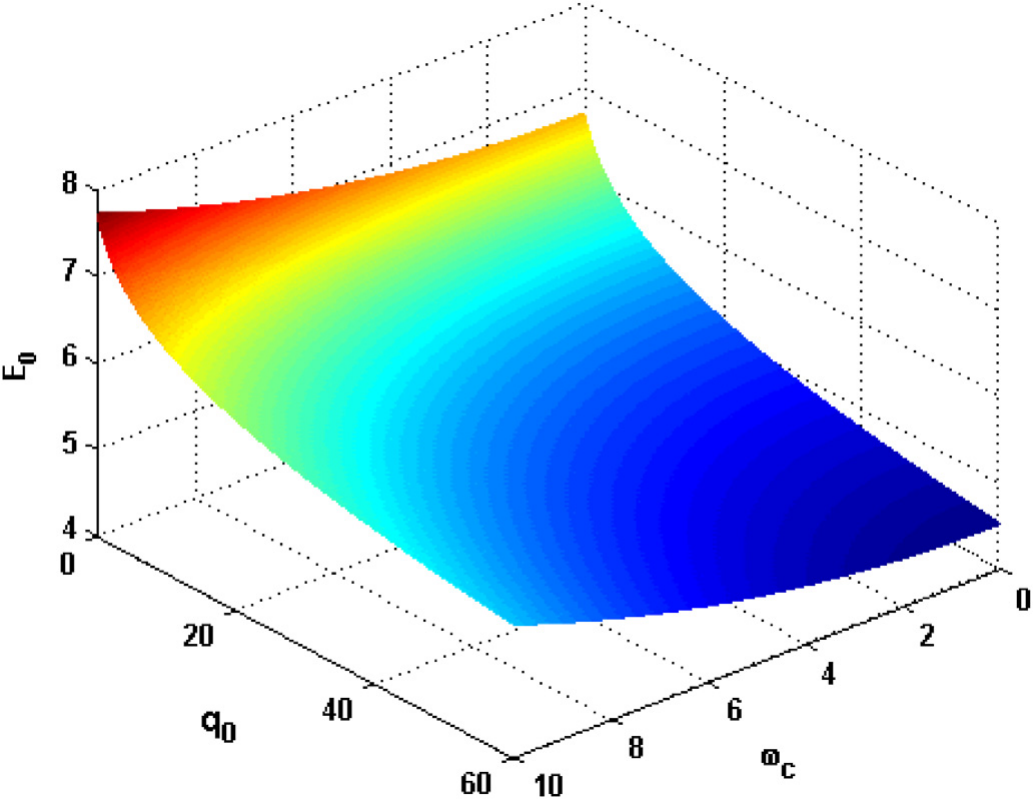
Relational curves of the GSE $E_{0}$ with the CF $\omega_{c}$ and the DCOW $q_{0}$ for α=0.1F=3, $V_{0}=4$ and R=5.

**Figure 4:**
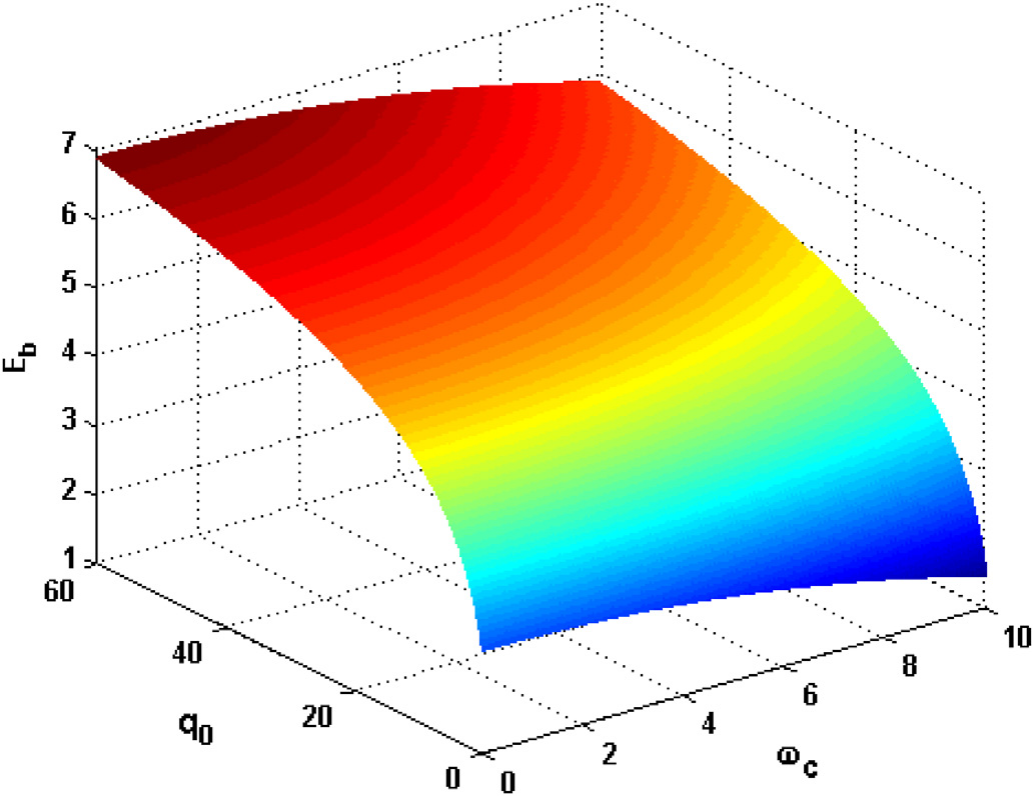
Relational curves of the GSBE $E_{b}$ with CF $\omega_{c}$ and DCOW $q_{0}$ for α=0.1F=3, $V_{0}=4$ and R=5.

**Figure 5:**
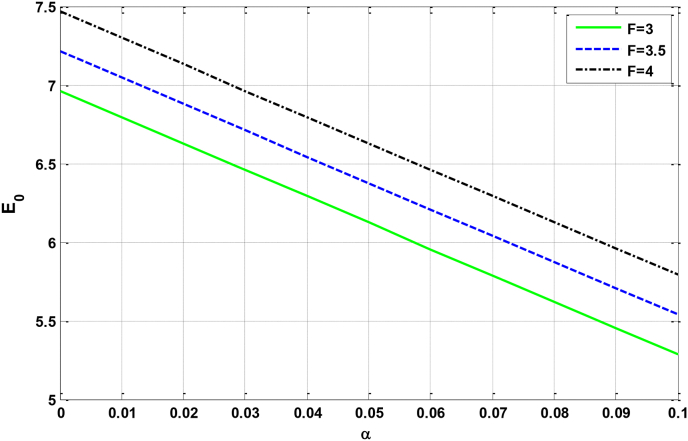
Relational curves of the GSE $E_{0}$ with the electron–phonon coupling strength α for $\omega_{c}=5$, $q_{0}=50$ , $V_{0}=4$ and R=5 with different values of F.

**Figure 6:**
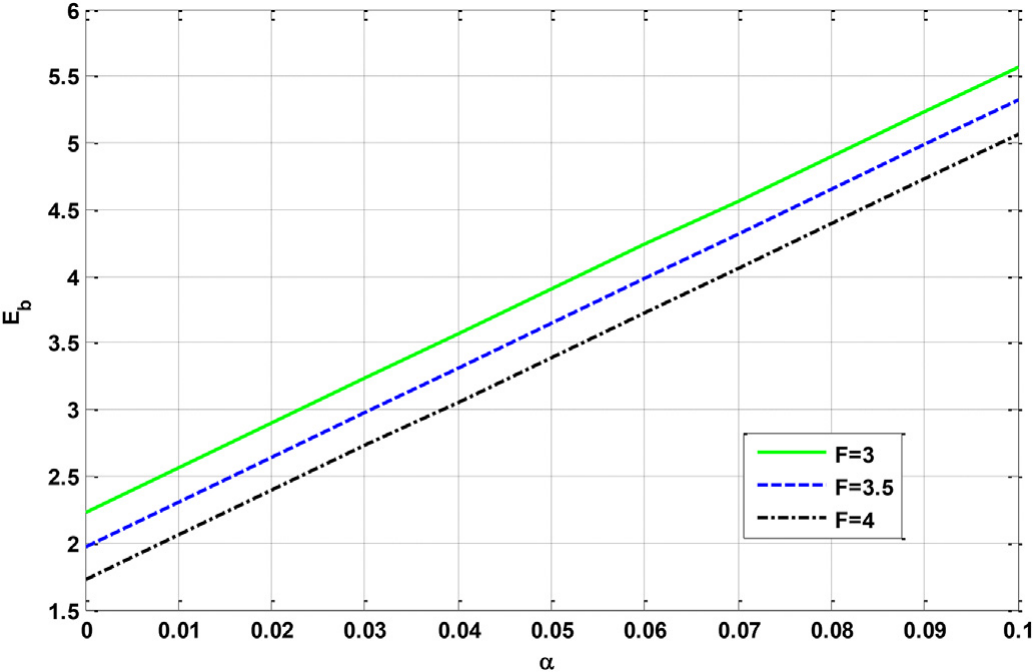
Relational curves of the GSBE $E_{b}$ with the electron–phonon coupling strength α for $\omega_{c}=5$$q_{0}=50$, $V_{0}=4$ and R=5 with different values of F.

In order to clearly demonstrate the influence of the EF F, the RAGCP R, CF $\omega_{c}$, the DCOW $q_{0}$ and electron–phonon coupling strength α parameters on the GSE $E_{0}$ and GSBE $E_{b}$, numerical computations will be performed. The numerical results are presented in Figures 1, 2, 3, 4, 5, and 6.

Figure 1 depicts the GSE as a function of RAGCP and EF F for $q_{0}=50$, $\omega_{c}=5$, $V_{0}=4$ and α=0.1. It can be seen that the GSE increases with EF. This is because the increase in EF leads to the increase in energy of the electrons causing them to interact with more phonons. Thus, the GSE is raised. From another perspective, since applying an external EF is equivalent to electrons’ confinement, which makes the electrons wave function overlapping stronger. Afterwards the electron-phonon interactions will be lifted. As a result, the GSE increases with increasing the EF. This result is in agreement with the one of [44]. In Figure 1, it is also seen that the GSE decreases with decreasing the RAGCP, displays an asymptotic profile and eventually conforms to a limiting value. The reason for this decrease is that, the RAGCP gives negative contribution to the GSE. Hence, we can obtain the results mentioned above.

In Figure 2, we can see that the GSBE $E_{b}$ is an aggrandizing function with decreasing the RAGCP R, whereas it is a diminishing function of the EF F. The physical reason is that, with the reduction of RAGCP, the spatial overlap between an electron and a phonon is raised. Thus, the thermal motion energy of electrons and the electron-phonons interaction, which take phonons as the medium, are lifted because the range of particle motion becomes restricted. Consequently, the GSBE of polaron increases with bringing down the RAGCP. This is in agreement with the work of [45]. It is also seen that the GSBE is decreasing functions of EF. However, the last term in Eq. (16) is the contribution from the EF to the GSBE, which is a negative value. Thus, the GSBE will decrease with increasing EF and it's according to Reference [46].

Figure 3 plots the GSE as a function of CF $\omega_{c}$ and DCOW $q_{0}$ with α=0.1 F=3, $V_{0}=4$ and R=5. It can be seen that the GSE increases with the CF. From the formula of $\omega_{c}=\frac{eB}{m}$, one can see that GSE will lift with rising the MF B. With the increase of MF, the electron energy and the energy of electron–phonon interaction are increased due to the presence of the MF. Therefore the GSE increases with rising CF. From another perspective, since the presence of the MF is similar to a source of additional confinement on the electrons, which leads to greater electron wavefunction overlapping with each other. Resulting the electron energy lifted and makes the electrons interact with more phonons, the electron–phonon interactions will be enhanced, thus the GSE lift with rising CF. This result has also been obtained by [13]. It can also be observed that the GSE decreases with increasing DCOW. As can be seen in last term in Eq. (14) we let's see that the DCOW gives negative contribution to the GSE. Thus, the rising DCOW bringing down the GSE. G. A. Farias, W. B. da Costa, and F. M. Peeters [47] highlighted that the GSE is the decreasing function of the Debey cut-off frequency this result is in accordance with our result. Also, we tried show that the electron interacting with an acoustic phonon branch via the deformation potential is an object whose complexity is going far beyond the optical phonon [48].

In Figure 4 we plot the GSBE as a function of CF $\omega_{c}$ and DCOW $q_{0}$ with α=0.1, F=3, $V_{0}=4$ and R=5. It can be seen that the GSBE increases with DCOW $q_{0}$. It is because the scattering rate of phonon by electron decreases with rising phonon frequency. From the formula of $\omega_{q}=s.q$ one can notice that the increasing piezo-acoustic phonon frequency strongly depends on the raising of DCOW. Moreover, the piezo-acoustic phonons with larger DCOW is less likely to be scattered by electrons. Therefore, electron-phonon interaction strength will lift with increasing the DCOW. In addition, the contribution from DCOW to the GSBE is positive. Consequently GSBE is increased with rising DCOW. In addition, one can find from Figure 4 that the GSBE decreases with increasing the CF. This is because the second term in Eq. (16) is the contribution from the CF to the GSBE, which is a negative value. Consequently the GSBE reduce with rising CF.

From Figure 5 we can observe that: the GSE is reduced with the increase of the electron–phonon coupling strength. This behavior can be justified by the fact that, the fifth term in Eq. (14) contains the contribution of the electron–phonon coupling strength who have a negative value. For this reason, the GSE will reduce with increasing electron–phonon interaction strength.

In Figure 6, we can see that the GSBE $E_{b}$ is an increase function of the electron–phonon coupling strength. The physical origin is that, the larger the electron-phonon coupling strength is, the stronger the electron-phonon interaction is. As a result of it, the GSBE is lifted with increasing the electron–phonon coupling strength. This outcome is in accordance with that of [49].

## 4 Conclusion

Based on the Lee-Low-Pines unitary transformation and linear combination operation methods, we have investigated the GSE and GSBE of a weak-coupling piezoelectric polaron in the AGCPQW in presence of MF and EF. It is found that: (1) the GSE is an increasing function of the EF and the CF, whereas it is a diminishing one of the RAGCP, the DCOW and the electron–phonon coupling strength. (2) The GSBE is an increasing function of the DCOW and the electron–phonon coupling strength. It is also an expanding function with decreasing the RAGCP, whereas it is a decayed one of the EF and CF. We found four parameters of controlling GSE and GSBE of a weak-coupling piezoelectric polaron such as: CF, the EF, the RAGCP, the DCOW and the electron–phonon coupling strength. This open potential application in nanostructure devices.

## Declarations

### Author contribution statement

J. FOTUE: Conceived and designed the experiments; Wrote the paper.

F. MANFOUO, I. NSANGOU: Performed the experiments; Contributed reagents, materials, analysis tools or data; Wrote the paper.

M.F.C. FOBASSO: Analyzed and interpreted the data; Contributed reagents, materials, analysis tools or data.

### Funding statement

This research did not receive any specific grant from funding agencies in the public, commercial, or not-for-profit sectors.

### Data availability statement

No data was used for the research described in the article.

### Declaration of interests statement

The authors declare no conflict of interest.

### Additional information

No additional information is available for this paper.
